# Monetary Diet Cost is Associated with not only Favorable but also Unfavorable Aspects of Diet in Pregnant Japanese Women: The Osaka Maternal and Child Health Study

**DOI:** 10.4137/EHI.S2508

**Published:** 2009-05-12

**Authors:** Kentaro Murakami, Yoshihiro Miyake, Satoshi Sasaki, Keiko Tanaka, Yukihiro Ohya, Yoshio Hirota, Osaka Maternal and Child Health Study Group

**Affiliations:** 1 Department of Social and Preventive Epidemiology, School of Public Health, the University of Tokyo, Tokyo, Japan; 2 Department of Public Health, Faculty of Medicine, Fukuoka University, Fukuoka, Japan; 3 Division of Allergy, Department of Medical Specialties, National Center for Child Health and Development, Tokyo, Japan; 4 Department of Public Health, Osaka City University School of Medicine, Osaka, Japan; 5 Other members of the Osaka Maternal and Child Health Study Group are listed in the Acknowledgments. Email: kenmrkm@m.u-tokyo.ac.jp

**Keywords:** diet cost, nutrient intake, food intake, pregnant women, Japan

## Abstract

While several observational studies in European countries have shown that higher monetary diet cost is associated with healthier diets, information on the relationship of cost to diet quality in other countries is sparse, including Japan. This cross-sectional study examined the association between monetary diet cost and dietary intake in a group of pregnant Japanese women. Subjects were 596 pregnant Japanese housewives. Dietary intake was estimated using a validated, self-administered, comprehensive diet history questionnaire. Monetary diet cost was calculated using retail food prices. Values of monetary diet cost and nutrient and food intake were energy-adjusted using the density method. Monetary diet cost was associated positively with the intake of protein, total fat, saturated fatty acids, dietary fiber, cholesterol, sodium, potassium, calcium, magnesium, iron, vitamins A, D, E, C, and folate, and inversely with that of carbohydrate. For foods, cost was associated positively with the intake of potatoes, pulses and nuts, fish and shellfish, meat, dairy products, vegetables, and fruits, and inversely with that of rice and bread. No association was seen for noodles, confectioneries and sugars, fats and oils, or eggs. Cost was also associated inversely with dietary energy density. In conclusion, monetary diet cost was associated with not only favorable aspects of diet, including a higher intake of dietary fiber, key vitamins and minerals, fruits, and vegetables and lower dietary energy density, but also unfavorable aspects, including a higher intake of fat and sodium and lower intake of carbohydrate and rice, in a group of pregnant Japanese women.

## Introduction

While food choice is influenced by a large number of factors,^1^ the price of food is an important determinant.^2^,^3^ Generally, energy-dense and nutrient-dilute foods such as cereals, fats and oils, and sugar and confectioneries provide dietary energy at lowest cost.^4^–^9^ Conversely, the cost per calorie of energy-dilute and nutrient-dense foods, including fish and shellfish, vegetables, and fruits is much higher.^4^–^9^ If healthier foods cost more then so too will healthier diets, and several observational studies conducted in European countries have in fact shown that healthful diets are more expensive than less healthful diets,^4^–^9^ albeit that the currently available procedure for estimating monetary diet cost is based on the assumption that all foods are purchased and then prepared and consumed at home, and hence cannot consider the monetary cost of eating out.^10^ Given the important role of nutrition in promoting health,^11^,^12^ the positive association between monetary diet cost and diet quality may explain, at least in part, the socioeconomic gradient in health widely observed in Western countries, with higher socioeconomic position associated with better health.^13^–^15^

However, information on the relationship of monetary diet cost to diet quality in other countries is sparse, including Japan. Considering the unclear or even inverse association between socioeconomic position and health status observed in several Japanese populations,^16^–^18^ the association between monetary diet cost and diet quality may differ between European countries and Japan. In a study of Japanese female dietetic students, for example, monetary diet cost was not necessarily associated with healthier dietary intake patterns.^19^ This finding may have been confounded by a lack of information on whether the participants bought their food themselves, however, given that the majority (89%) lived with their family.^19^ Because the relationship between monetary diet cost and diet quality is an important public health issue, further investigation in Japanese people is clearly needed. Additionally, the association between monetary diet cost and dietary intake has not been investigated among women during pregnancy, a nutritionally important lifestyle stage, even in Western countries.

The present cross-sectional study examined the association between monetary diet cost and dietary intake in a group of pregnant Japanese women using baseline data from the Osaka Maternal and Child Health Study (OMCHS). We selected only housewives who did not habitually eat out and who lived in nuclear families in non-agricultural societies for accurate estimation of monetary diet cost. We hypothesized that monetary diet cost is associated with both favorable and unfavorable aspects of diet.

## Methods

### Subjects

The subjects in the present analysis were participants in the baseline survey of the OMCHS, an ongoing prospective cohort study, the main purpose of which was to investigate preventive and risk factors for maternal and child health problems. Details of the OMCHS have been published elsewhere.^20^,^21^ Briefly, all pregnant women in Neyagawa City, Osaka Prefecture (metropolis in Japan with a total population of approximately 8.8 million), were recruited between November 2001 and March 2003. Of 3639 eligible women, 627 (17.2%) took part in the study. An additional 375 pregnant women living in other neighborhood areas were enrolled in the study between December 2001 and November 2003, giving a total of 1002 pregnant women completing the baseline survey. Written informed consent was obtained from each woman. The protocol of the OMCHS was approved by the ethics committees of Osaka City University School of Medicine, Japan.

For the present analysis, we excluded women living in an expanded family (n = 127), those working outside the home (n = 288), and those who dined out 16 times or more during the preceding month (i.e. four times or more per week) (n = 118). Because some women fell into more than one exclusion category, the final analysis sample comprised 596 women.

### Measurements

Baseline assessment of the OMCHS included a set of two self-administered questionnaires. The participants mailed the answered questionnaires to the data management center. Research technicians completed missing or illogical data by telephone interview.

One of the self-administered questionnaires was a validated, self-administered, comprehensive diet history questionnaire (DHQ) that assesses dietary habits during the preceding month. Details of the DHQ’s structure, calculation of dietary intake, and validity for commonly studied nutritional factors have been published elsewhere.^22^–^25^ Briefly, the DHQ is a 16-page structured questionnaire which asks about the consumption frequency and portion size of selected foods commonly consumed in Japan as well as general dietary behavior and usual cooking methods.^22^ Estimates of daily intake for foods (150 items in total), nutrients, and energy were calculated using an ad hoc computer algorithm for the DHQ,^22^,^25^ based on the Standard Tables of Food Composition in Japan.^26^

Monetary diet cost (Japanese yen/day) was calculated by multiplying the amount of each food estimated from the DHQ (g/day) by the estimated price of the food (Japanese yen/g) and summing the products (1 Japanese yen = 0.0077 pound sterling = 0.0085 euros = 0.0110 US dollars in February 2009). Detailed descriptions of the cost calculation method as well as the monetary cost of each food have been published elsewhere.^19^,^27^ Briefly, the price of each food was determined based on the National Retail Price Survey 2004.^28^ For foods whose prices were not published in the survey (13 items), prices were taken from the websites of a nationally distributed supermarket (Seiyu, Tokyo, Japan) or fast-food restaurant (McDonalds, Tokyo, Japan and Mister Donut, Tokyo, Japan) chains. Alcoholic beverages (six items), non-caloric beverages (four items), and water (three items) were excluded from calculation.^9^ Costs of combined foods such as pizza were calculated using the prices of frozen equivalents.^8^ Costs were estimated based on the assumption that all foods were purchased and then prepared and consumed at home.^10^

To minimize the influence of dietary misreporting, an ongoing controversy in studies that collect dietary information using self-report instruments,^29^ values of monetary diet cost as well as intakes of nutrients and foods were energy-adjusted using the density method (i.e. monetary diet cost per 1000 kcal of energy, percentage of energy for energy-providing nutrients, and amount per 1000 kcal of energy for other nutrients and foods). Dietary energy density (kcal/g) was calculated based on foods only (128 items, excluding 22 beverages), as described elsewhere.^30^ In a previous study of 92 Japanese women, Pearson correlation coefficient between the DHQ and 16-day weighed dietary records were 0.57 for monetary diet cost, suggesting satisfactory validity of the DHQ in terms of monetary diet cost.^27^

The other self-administered questionnaire elicited information on potential confounding factors, including age (28 years or less, 29–31 years, and 32 years or more), gestational age (14 weeks or less, 15–20 weeks, and 21 weeks or more), parity (nulliparous and multiparous), cigarette smoking (never, former, and current), changes in diet in the previous one month (none or few, slight, and substantial), physical activity (low; and medium or high), partner’s education [low (12 years or less), medium (13–14 years), and high (15 years or more)], education [low (12 years or less), medium (13–14 years), and high (15 years or more)], and household income as calculated considering differences in household size and composition^31^ [divided into approximate tertiles: low (2,499,999 yen/year or less), medium (2,500,000–3,099,999 yen/year), and high (3,100,000 yen/year or more)].

### Statistical analysis

All statistical analyses were performed using SAS statistical software version 9.1 (SAS Institute Inc., Cary, NC, USA). Using the PROC GLM procedure, linear regression models were constructed to examine the association of monetary diet cost with dietary intake of selected nutrients and foods. For analyses, subjects were categorized into quartiles according to monetary diet cost. Mean (and standard error) values of nutrient and food intake were calculated by quartile of monetary diet cost with adjustment for potential confounding factors, including age, gestational age, parity, cigarette smoking, changes in diet in the previous month, physical activity, partner’s education, education, and household income. Tests for trends were performed by modeling the median value of each quartile category as a continuous variable. All reported *P*-values are two-tailed, and a *P*-value of <0.05 was considered statistically significant.

## Results

Dietary characteristics of the 596 women are shown in Table 1. Mean monetary diet cost was 458.9 Japanese yen/1000 kcal, while mean percentages of intake of protein, total fat, and carbohydrate were 13.4%, 30.0%, and 55.6% of energy, respectively. Selected characteristics by quartile of monetary diet cost are shown in Table 2. Women in the higher quartiles of cost tended to be never smokers and have changes in diet in the previous month, a partner with higher education, higher education, and higher household income.

Dietary characteristics by quartile of monetary diet cost are shown in Table 3. At the nutrient level, monetary diet cost was associated positively with the intake of protein, total fat, saturated fatty acids, dietary fiber, cholesterol, sodium, potassium, calcium, magnesium, iron, vitamins A, D, E, and C, and folate, and inversely with that of carbohydrate. At the food level, monetary diet cost was associated positively with the intake of potatoes, pulses and nuts, fish and shellfish, meat, dairy products, vegetables, and fruits, and inversely with that of rice and bread. No association was seen for noodles, confectioneries and sugars, fats and oils, or eggs. Further, monetary diet cost was inversely associated with dietary energy density.

## Discussion

Consistent with a previous study of Japanese female dietetic students,^19^ we found that monetary diet cost was associated with not only favorable aspects of diet such as a higher intake of dietary fiber, key vitamins and minerals, and fruits and vegetables and lower dietary energy density, but also unfavorable aspects of diet such as a higher intake of fat and sodium and lower intake of carbohydrate and rice in a group of pregnant Japanese women. These findings somewhat conflict with those of previous Western studies, in which monetary diet cost has been consistently associated with healthier diets, including higher intake of key micronutrients, fruits, and vegetables, lower intake of fats and sweets, and lower dietary energy density.^4^–^9^

For Japanese people, rice is a staple food which is consumed at almost every meal, accompanied by a main and several side dishes consisting mainly of fish and shellfish, meat, egg, vegetables, and pulses. Rice is relatively inexpensive compared with the component foods of such main and side dishes in Japan.^19^ We previously speculated that persons with a limited budget for foods would mainly consume rice, with a relatively restricted amount or variety of main and side dishes, while those with a larger food budget would have an increased amount or variety of main and side dishes and decreased consumption of rice.^19^ This hypothesis may be supported by the decreasing consumption of rice and increasing consumption of other foods such as meat, vegetables, fish and shellfish, and pulses^32^ seen with the increase in the Gross National Product of Japan^33^ from 1955 to 2000.^19^

With regard to the unfavorable aspects of diet at the nutrient level, the higher intake of total fat, saturated fatty acids, and cholesterol and lower intake of carbohydrate seen with increasing monetary diet cost appeared to be due to lower rice consumption (and dietary intake patterns associated with lower rice intake) with increasing monetary diet cost; in apparent support of this, rice intake was in fact negatively associated with the intake of total fat (Pearson correlation coefficient: −0.52), saturated fatty acids (−0.48), and cholesterol (−0.19) and positively with carbohydrate intake (0.44). The higher intake of sodium seen with increasing monetary diet cost may be due to higher intake of vegetables, meat, fish and shellfish, and pulses and nuts, because in Japan these foods are usually accompanied by seasonings with a salty taste, such as salt, soy sauce, miso (fermented soybean paste), and dressings. Intake of these foods was in fact positively associated with sodium intake in the present study (Pearson correlation coefficients: 0.33, 0.12, 0.30, and 0.35, respectively).

Several limitations of the study warrant mention. First, the study subjects were selected pregnant Japanese housewives. Additionally, response rate for the 627 women living in Neyagawa City was only 17.2%, and could not be calculated for the remaining 375 women living in other areas because the exact number of eligible subjects among the sources from which they recruited was not available. Thus, the present results could not be extrapolated to other populations.

Although the validity of the DHQ appears reasonable with regard to commonly studied nutritional factors,^22^–^25^ it was not specifically designed to measure monetary diet cost. This limitation is common to other studies on this topic.^4^–^9^,^19^ Additionally, food prices were derived from the National Retail Price Survey and websites of nationally distributed supermarket and fast food restaurant chains. As this procedure gives only an approximation of actual diet costs, the results of the present study should be interpreted with caution, although a similar methodology has been used in all previous observational studies.^4^–^9^,^19^ Nevertheless, the satisfactory validity of this DHQ regarding monetary diet cost against 16-day weighed dietary records as described above may provide some reassurance.^27^

In conclusion, monetary diet cost was associated with not only favorable but unfavorable aspects of diet in a group of pregnant Japanese women. Because the relation between monetary diet cost and diet quality is an important public health topic, further research is needed to investigate whether the association found in this specific group of subjects would also be observed in other Japanese populations.

## Figures and Tables

**Table 1.** Dietary characteristics of the subjects (n = 596).^a^

**Variable**	
Monetary diet cost (Japanese yen/1000 kcal)^b^	458.9 ± 72.7
Nutrient intake	
Protein (% of energy)	13.4 ± 2.0
Total fat (% of energy)	30.0 ± 5.5
Saturated fatty acids (% of energy)	8.5 ± 2.0
Carbohydrate (% of energy)	55.6 ± 6.3
Dietary fiber (g/1000 kcal)	6.5 ± 1.8
Cholesterol (mg/1000 kcal)	164.5 ± 59.5
Sodium (mg/1000 kcal)	2039 ± 437
Potassium (mg/1000 kcal)	1151 ± 270
Calcium (mg/1000 kcal)	294.5 ± 97.8
Magmesium (mg/1000 kcal)	121.3 ± 26.2
Iron (mg/1000 kcal)	3.66 ± 0.84
Vitamin A (μg retinol equivalents/1000 kcal)	341.3 ± 317.8
Vitamin D (mg/1000 kcal)	3.34 ± 1.78
Vitamin E (mg alpha-tocopherol/1000 kcal)	4.25 ± 0.91
Vitamin C (mg/1000 kcal)	58.6 ± 28.5
Folate (μg/1000 kcal)	157.4 ± 49.2
Food intake (g/1000 kcal)	
Rice	133.9 ± 56.6
Bread	38.8 ± 22.5
Noodles	43.5 ± 32.4
Potatoes	15.2 ± 11.4
Confectioneries and sugars	33.8 ± 17.3
Fats and oils	13.6 ± 6.2
Pulses and nuts	26.2 ± 16.9
Fish and shellfish	26.1 ± 13.5
Meat	32.5 ± 15.2
Eggs	17.9 ± 13.0
Dairy products	103.8 ± 67.5
Vegetables	115.5 ± 67.4
Fruits	94.8 ± 92.7
Dietary energy density (kcal/g)	1.38 ± 0.20

a Values are mean ± standard error.

b 1 Japanese yen = 0.0077 pound sterling = 0.0085 euros = 0.0110 U.S. dollars in February 2009.

**Table 2.** Selected characteristics according to quartile category of monetary diet cost (n = 596).^a^

**Variable**	**Qartile 1 (n = 149)**	**Quartile 2 (n = 149)**	**Quartile 3 (n = 149)**	**Quartile 4 (n = 149)**	**P^b^**
Monetary diet cost (Japanese yen/1000 kcal)^c^					
Median	379.6	434.1	475.3	536.6	
Range	223.6–412.8	412.9–453.5	453.6–501.4	501.5–829.2	
Age					0.18
28 years or less	72 (48.3)	54 (36.2)	51 (34.2)	57 (38.3)	
29–31 years	33 (22.2)	53 (35.6)	43 (28.9)	48 (32.2)	
32 years or more	44 (29.5)	42 (28.2)	55 (36.9)	44 (29.5)	
Gestational age					0.41
14 weeks or less	50 (33.6)	58 (38.9)	51 (34.2)	53 (35.6)	
15–20 weeks	45 (30.2)	45 (30.2)	57 (38.3)	52 (34.9)	
21 weeks or more	54 (36.2)	46 (30.9)	41 (27.5)	44 (29.5)	
Parity					0.029
Nulliparous	54 (36.2)	70 (47.0)	57 (38.3)	78 (52.4)	
Multiparous	95 (63.8)	79 (53.0)	92 (61.7)	71 (47.7)	
Cigarette smoking					0.021
Never	88 (59.1)	111 (74.5)	101 (67.8)	106 (71.1)	
Former	20 (13.4)	19 (12.8)	28 (18.8)	20 (13.4)	
Current	41 (27.5)	19 (12.8)	20 (13.4)	23 (15.4)	
Changes in diet in the previous one month					0.0007
None or few	56 (37.6)	50 (33.6)	35 (23.5)	35 (23.5)	
Slight	61 (40.9)	66 (44.3)	76 (51.0)	64 (43.0)	
Substantial	32 (21.5)	33 (22.2)	38 (25.5)	50 (33.6)	
Physical activity					0.97
Low	101 (67.8)	81 (54.4)	91 (61.1)	98 (65.8)	
Medium or high	48 (32.2)	68 (45.6)	58 (38.9)	51 (34.2)	
Partner’s education					0.0020
Low (12 years or less)	69 (46.3)	68 (45.6)	58 (38.9)	50 (33.6)	
Medium (13–14 years)	30 (20.1)	25 (16.8)	20 (13.4)	25 (16.8)	
High (15 years or more)	50 (33.6)	56 (37.6)	71 (47.7)	74 (49.7)	
Education					<0.0001
Low (12 years or less)	64 (43.0)	61 (40.9)	46 (30.9)	38 (25.5)	
Medium (13–14 years)	58 (38.9)	60 (40.3)	61 (40.9)	63 (42.3)	
High (15 years or more)	27 (18.1)	28 (18.8)	42 (28.2)	48 (32.2)	
Household income					<0.0001
Low (2,499,999 yen/year or less)	76 (51.0)	55 (36.9)	45 (30.2)	45 (30.2)	
Medium (2,500,000–3,099,999 yen/year)	50 (33.6)	54 (36.2)	53 (35.6)	45 (30.2)	
High (3,100,000 yen/year or more)	23 (15.4)	40 (26.9)	51 (34.2)	59 (39.6)	

a Values are number of subjects (%) unless otherwise indicated.

b Mantel-Haenszel chi-square test.

c 1 Japanese yen = 0.0077 pound sterling = 0.0085 euros = 0.0110 US dollars in February 2009.

**Table 3.** Dietary characteristics according to quartile category of monetary diet cost (n = 596).^a^

**Variable**	**Qartile 1 (n = 149)**	**Qartile 2 (n = 149)**	**Qartile 3 (n = 149)**	**Qartile 4 (n = 149)**	**P for trend^b^**
Nutrient intake					
Protein (% of energy)	12.1 ± 0.1	13.2 ± 0.1	13.7 ± 0.1	14.6 ± 0.1	<0.0001
Total fat (% of energy)	27.8 ± 0.5	30.2 ± 0.4	30.8 ± 0.4	31.2 ± 0.4	<0.0001
Saturated fatty acids (% of energy)	7.5 ± 0.2	8.5 ± 0.2	8.9 ± 0.2	8.9 ± 0.2	<0.0001
Carbohydrate (% of energy)	58.5 ± 0.5	55.5 ± 0.5	54.6 ± 0.5	54.0 ± 0.5	<0.0001
Dietary fiber (g/1000 kcal)	5.6 ± 0.1	6.0 ± 0.1	6.5 ± 0.1	7.7 ± 0.1	<0.0001
Cholesterol (mg/1000 kcal)	149.1 ± 4.9	167.9 ± 4.9	173.7 ± 4.8	167.2 ± 4.9	0.012
Sodium (mg/1000 kcal)	1890 ± 36	1997 ± 36	2120 ± 36	2150 ± 36	<0.0001
Potassium (mg/1000 kcal)	937 ± 17	1087 ± 17	1191 ± 18	1391 ± 18	<0.0001
Calcium (mg/1000 kcal)	245.3 ± 7.2	286.0 ± 7.1	308.6 ± 7.1	338.2 ± 7.1	<0.0001
Magmesium (mg/1000 kcal)	106.8 ± 1.9	115.9 ± 1.9	123.4 ± 1.9	139.1 ± 1.9	<0.0001
Iron (mg/1000 kcal)	3.22 ± 0.06	3.50 ± 0.06	3.68 ± 0.06	4.21 ± 0.06	<0.0001
Vitamin A (μg retinol equivalents/1000 kcal)	230.9 ± 25.4	313.1 ± 25.0	343.3 ± 24.9	477.9 ± 25.0	<0.0001
Vitamin D (mg/1000 kcal)	2.59 ± 0.14	3.08 ± 0.14	3.52 ± 0.14	4.18 ± 0.14	<0.0001
Vitamin E (mg alpha-tocopherol/1000 kcal)	3.69 ± 0.07	4.09 ± 0.07	4.36 ± 0.07	4.85 ± 0.07	<0.0001
Vitamin C (mg/1000 kcal)	43.0 ± 2.1	50.4 ± 2.1	60.1 ± 2.0	80.8 ± 2.1	<0.0001
Folate (μg/1000 kcal)	133.0 ± 3.5	144.7 ± 3.5	157.4 ± 3.5	194.6 ± 3.5	<0.0001
Food intake (g/1000 kcal)					
Rice	169.1 ± 4.4	136.1 ± 4.3	123.7 ± 4.3	106.5 ± 4.3	<0.0001
Bread	44.7 ± 1.9	40.9 ± 1.9	36.3 ± 1.8	33.3 ± 1.9	<0.0001
Noodles	45.1 ± 2.7	48.1 ± 2.7	40.8 ± 2.7	39.9 ± 2.7	0.07
Potatoes	12.9 ± 0.9	14.5 ± 0.9	15.8 ± 0.9	17.8 ± 0.9	0.0001
Confectioneries and sugars	33.8 ± 1.5	33.7 ± 1.4	34.8 ± 1.4	32.6 ± 1.4	0.65
Fats and oils	13.8 ± 0.5	13.9 ± 0.5	13.3 ± 0.5	13.2 ± 0.5	0.30
Pulses and nuts	21.8 ± 1.4	23.0 ± 1.3	27.6 ± 1.3	32.3 ± 1.3	<0.0001
Fish and shellfish	18.5 ± 1.0	23.7 ± 1.0	29.3 ± 1.0	33.1 ± 1.0	<0.0001
Meat	24.8 ± 1.2	32.7 ± 1.2	33.1 ± 1.2	39.3 ± 1.2	<0.0001
Eggs	17.6 ± 1.1	19.3 ± 1.1	19.1 ± 1.1	15.7 ± 1.1	0.18
Dairy products	84.7 ± 5.5	109.3 ± 5.4	109.8 ± 5.4	111.3 ± 5.4	0.0016
Vegetables	73.8 ± 4.9	103.3 ± 4.8	119.6 ± 4.8	165.1 ± 4.8	<0.0001
Fruits	60.1 ± 7.4	78.0 ± 7.3	104.2 ± 7.2	136.9 ± 7.3	<0.0001
Dietary energy density (kcal/g)	1.48 ± 0.02	1.41 ± 0.02	1.36 ± 0.01	1.27 ± 0.02	<0.0001

a Values are means ± standard error. Adjustment was made for age (28 years or less, 29–31 years, and 32 years or more), gestational age (14 weeks or less, 15–20 weeks, and 21 weeks or more), parity (nulliparous and multiparous), cigarette smoking (never, former, and current), changes in diet previous one month (none or few, slight, and substantial), physical activity (low; and medium or high), and partner’s education [low (12 years or less), medium (13–14 years), and high (15 years or more)], education [low (12 years or less), medium (13–14 years), and high (15 years or more)], and household income [low (2,419,999 yen/year or less), medium (2,420,000–3,619,999 yen/year), and high (3,620,000 yen/year or more)].

b Test for linear trend in general linear regression.
